# Determination of Key Components in the *Bombyx mori* p53 Apoptosis Regulation Network Using Y2H-Seq

**DOI:** 10.3390/insects14040362

**Published:** 2023-04-05

**Authors:** Meixian Wang, Jiahao Wang, Ayinuer Yasen, Bingyan Fan, J. Joe Hull, Xingjia Shen

**Affiliations:** 1 Jiangsu Key Laboratory of Sericultural Biology and Biotechnology, College of Biotechnology, Jiangsu University of Science and Technology, Zhenjiang 212100, China; 2 Key Laboratory of Silkworm and Mulberry Genetic Improvement, Ministry of Agriculture and Rural Affairs, Sericultural Research Institute, Chinese Academy of Agricultural Sciences, Zhenjiang 212100, China; 3 USDA-ARS Arid Land Agricultural Research Center, Maricopa, AZ 85138, USA

**Keywords:** apoptosis, *Bombyx mori*, Bmp53, model, pathway, Y2H-Seq

## Abstract

**Simple Summary:**

Our study confirmed that Bmp53 can directly induce cell apoptosis and regulate the morphology and development of larvae during pupation. Y2H-Seq data led to the development of a model for apoptosis regulation with Bmp53 as a central node and the prediction that the silkworm Mdm2-like protein may be a key component of the apoptotic pathway. These results provide the initial molecular foundation for elucidating apoptosis regulation in silkworms and assessing the various biological processes regulated by the Bmp53 interactome.

**Abstract:**

The apoptosis pathway is highly conserved between invertebrates and mammals. Although genes encoding the classical apoptosis pathway can be found in the silkworm genome, the regulatory pathway and other apoptotic network genes have yet to be confirmed. Consequently, characterizing these genes and their underlying mechanisms could provide critical insights into the molecular basis of organ apoptosis and remodeling. A homolog of p53, a key apoptosis regulator in vertebrates, has been identified and cloned from *Bombyx mori* (Bmp53). This study confirmed via gene knockdown and overexpression that Bmp53 directly induces cell apoptosis and regulates the morphology and development of individuals during the metamorphosis stage. Furthermore, yeast two-hybrid sequencing (Y2H-Seq) identified several potential apoptotic regulatory interacting proteins, including the MDM2-like ubiquitination regulatory protein, which may represent an apoptosis factor unique to Bmp53 and which differs from that in other Lepidoptera. These results provide a theoretical basis for analyzing the various biological processes regulated by Bmp53 interaction groups and thus provide insight into the regulation of apoptosis in silkworms. The global interaction set identified in this study also provides a basic framework for future studies on apoptosis-dependent pupation in Lepidoptera.

## 1. Introduction

Apoptosis is a highly regulated process that leads to the death and elimination of individual cells with few negative effects on surrounding tissue. As such, its role in the removal of damaged or malfunctioning cells is critical in the development of many diseases and in tissue replacement [1,2]. In addition to being a model lepidopteran insect, *Bombyx mori* (*B. mori*) has also been used to provide insights into basic medical research [3]. Initially, research on silkworm apoptosis lagged far behind studies conducted in other organisms; however, recent studies have focused on identifying the internal (e.g., hormones and/or lymph) and external factors (e.g., chemicals, UV light, and viruses) that impact its cellular induction. Similarly, significant effort has gone into understanding how apoptosis affects tissue development (e.g., wings) and determining the conditions that drive morphological changes and/or impact tissue organization, such as those frequently seen in the intestine and silk gland [4,5,6,7,8,9]. While progress has also been made in identifying and characterizing the regulatory mechanisms underlying apoptosis-related genes [10,11,12] and the availability of the *B. mori* genome has facilitated functional studies, the silkworm apoptosis network has yet to be as fully developed as that of *Drosophila melanogaster*.

The p53 protein is a central regulator of the cellular stress response and may be involved in *B. mori* apoptosis. It is activated by various stress signals and initiates specific cellular responses according to the nature of the stress, the cell type, and the cell environment [13,14]. It is a key tumor suppressor, and one of the most commonly mutated genes in human cancer. Homologs of p53 have been identified in many insect groups, including the Homoptera, Hymenoptera, Coleoptera, Lepidoptera, and Diptera [15]. The insect proteins identified to date are structurally similar to vertebrate p53 and contain a transactivation domain (TAD) and adjacent proline-rich regions, a DNA-binding domain (DBD), and an oligomerization domain (OD) [16]. The *p53* homolog in *B. mori* (*Bmp53*) was successfully cloned in 2011 [15], and its role in regulating apoptosis was confirmed by studies on the anti-viral immune response of silkworms to *B. mori* nuclear polyhedrosis virus [17,18]. Further, apoptosis mechanisms have been shown to be critical for *B. mori* development and metamorphosis [19]. Surprisingly, Bmp53 appears to be more closely related to vertebrate p53s than those of *Drosophila* or *Caenorhabditis elegans* [20]. Studies into the regulatory mechanisms underlying Bmp53-mediated apoptosis in *B. mori* can thus expand our understanding of critical points in the silkworm lifecycle as well as provide crucial insights into the function of p53 in other biological contexts.

Most biological processes proceed by recruiting diverse proteins to form a multimeric protein complex. Numerous methodological approaches, such as immunoprecipitation, pull-down assays, and yeast two-hybrid (Y2H) assays, have been developed to elucidate the protein–protein interactions that comprise protein complexes [21]. Among them, the Y2H system has been the most effective method for large-scale screens in both vertebrates and invertebrates [22,23]. Despite its utility, the traditional Y2H system is limited by low plasmid copy number and high costs associated with clonal sequencing. Here, we used a Y2H-Seq method that combined Y2H with next-generation sequencing [24,25]. By optimizing this approach, we screened the *Bmp53* interactome for potential roles in regulating apoptosis. Based on these results, we developed a model for Bmp53-mediated regulation of apoptosis, which impacts silkworm metamorphosis and development. These data provide a molecular basis for understanding the mechanisms underlying apoptosis regulation in silkworms.

## 2. Materials and Methods

### 2.1. Cells and Animals

*B. mori* ovary-derived BmN cells were cultured in TC-100 insect medium supplemented with 10% FBS, 100 units/mL penicillin, and 100 mg/mL streptomycin at 28 °C. HEK293T cells were cultured in DMEM supplemented with 10% FBS, 2 mM glutamine, 100 units/mL penicillin, and 100 mg/mL streptomycin. Cells were maintained at 37 °C in a humidified atmosphere with 5% CO_2_.

The *B. mori p50* strain was bred and preserved by the Sericulture Research Institute of the Chinese Academy of Agricultural Sciences. Diapause-terminated eggs (induced by HCl exposure) were incubated in indoor natural light at 25 °C. After hatching, silkworm larvae were reared with fresh mulberry leaves at 25 ± 1 °C.

### 2.2. Bmp53 Overexpression in Cells

The *Bmp53* open-reading frame (ORF; BankIt Submission 2651619) was PCR amplified using Bmp53-F and Bmp53-R primers (Table 1) from SunYA Inc. (Hangzhou, China) and an amplification program consisting of an initial 98 °C for 3 min followed by 95 °C for 3 min, 35 cycles at 95 °C for 30 s, 55 °C for 20 s, 72 °C for 1 min, and finally 72 °C for 5 min. The products were then cloned into the BamHI/XhoI sites of pcDNA3.1-3xFLAG-c (Youbia, Changsha, China) (Supplementary Figure S1A). HEK293T cells stably expressing pcDNA3.1-3xFLAG-c-Bmp53 were established using EntransterTM-H4000 reagent (Engreen, Beijing, China). The amplified Bmp53 ORF was similarly cloned using Bmp53-F and Bmp53-R(CC) primers (Table 1) into the BamHI/XhoI sites of pIZ-EGFP-V5-His (Invitrogen, Carlsbad, CA, USA) (Supplementary Figure S1B). Stable expression of pIZ-EGFP-Bmp53-V5-His was likewise established using EntransterTM-H4000 reagent (Engreen) in BmN cells, a *B. mori* ovarian cell line frequently used for functional analysis of heterologously expressed genes. All transient transfections were performed according to the manufacturer’s protocol. Fluorescent cells images were processed using Adobe Photoshop v 24.2 (Adobe, San Jose, CA, USA).

Bmp53 fusion proteins were separated via 4–20% SDS PAGE for 60 min at 300 mA and then transferred to a PVDF membrane for immunodetection using murine-derived anti-FLAG (Cat#F1804, Sigma, St. Louis, MO, USA) or anti-EGFP (Cat#ATM1006, NULEN BIOTECH) antibodies. Detection of Bmp53-induced cell apoptosis was performed approximately 96 hrs after transfection using a TUNEL BrightRed Apoptosis Detection Kit (Cat#A113, Vazyme, Nanjing, China). Caspase activity was assayed using caspase 3 and caspase 8 activity assay kits (Cat#BC3830 and Cat#BC3850, Solarbio, Beijing, China) according to the manufacturer’s protocol. Statistical analyses were performed using the student *t*-test in GraphPad Prism v 9 (GraphPad Software, Boston, MA, USA).

### 2.3. Bmp53 Knockdown in Silkworm Larvae

dsRNA primers (Table 1) containing a T7 promoter sequence for amplifying Bmp53 and GFP (green fluorescent protein) were designed according to the NCBI database and the online software SnapDragon-dsRNA Design (https://www.flyrnai.org/snapdragon (accessed on 6 April 2022)). dsRNAs were synthesized and purified using a T7 RiboMAXTM Express RNAi System (Promega, Madison, WI, USA). All the samples were stored at −20 °C after determining the concentrations. For RNAi experiments, 0.5 µg Bmp53 dsRNA (2.5 µL of 200 ng/µL) was injected into the basal side of 6-day-old fifth instar larvae using a 5 µL microinjector (10 larvae per group with 3 replicates each). The same quantity of dsGFP was injected into control larvae. Target transcript knockdown was confirmed via qPCR using primers q53-F and q53-R (Table 1) on a QuantStudio 6 Flex Real-Time PCR System (Applied Biosystems, Foster, CA, USA) with the following conditions: 95 °C for 30 s, 40 cycles at 95 °C for 10 s, 60 °C for 30 s or 95 °C 15 s, 60 °C for 60 s, and then 95 °C for 15 s. Statistical analyses of larval phenotypes was performed using the Chi-square test in GraphPad Prism.

### 2.4. Y2H-Seq Assay

For the Y2H-Seq assay, we used a pGADT7 vector system (Ruiyuan Biotechnology Co. Ltd., Nanjing, China) to construct a BmN yeast expression library. Total RNA was extracted from ~2 × 10^8^ BmN cells using Trizol, and mRNAs were purified with oligo-(dT) magnetic beads. The pGADT7 expression vectors were generated using cDNAs synthesized from 500 ng DNAase-treated mRNA, and the *Bmp53* sequence was inserted into the pGBKT7 bait vector via homologous recombination. pGADT7 vectors were introduced into the Y187 strain of *Saccharomyces cerevisiae*, and pGBKT7-Bmp53 was used to verify self-activation. The AH109 yeast strain, containing a pGBKT7-Bmp53 decoy plasmid, was used as a receptor. Bmp53-interacting proteins were screened using the Y2H mating protocol according to the manufacturer’s instructions. The BmN library was screened using plates coated with SD-TLH+10 mM 3AT. Positive clones were PCR amplified from yeast cells, and the resulting products were subjected to NGS sequencing (Ruiyuan Biotechnology Co. Ltd., Nanjing, China). BLAST analyses were performed using the GenBank nr database.

### 2.5. Y2H-Seq Data Analysis

The NGS “*Bombyx mori* Bmp53-Interacting Protein High-Throughput Sequencing Screen Library Results” data (~10 GB) were annotated according to SilkDB 3.0 (SilkDB 3.0; bioinfotoolkits.net). GO (Gene Ontology) enrichment, KEGG (Kyoto Encyclopedia of Genes and Genomes) pathway analysis, and apoptosis-specific expression analyses were performed with TBtools [26]. The list of potential Bmp53-interacting proteins was analyzed with STRING (https://cn.string-db.org/ (accessed on 16 April 2022)), with a focus on apoptosis-related proteins and E3 ubiquitin ligase Mdm2. The Y2H-Seq data also included 10 transcription factors (Supplementary Data S1). To assess their potential role in Bmp53 function/interactions, a 2000-bp region of the *B. mori* genome directly upstream of the *Bmp53* start site was identified using TBtools [26].

## 3. Results

### 3.1. Bmp53 Overexpression in Both Mammalian and Insect Cells InducesApoptosis

**Figure 1:**
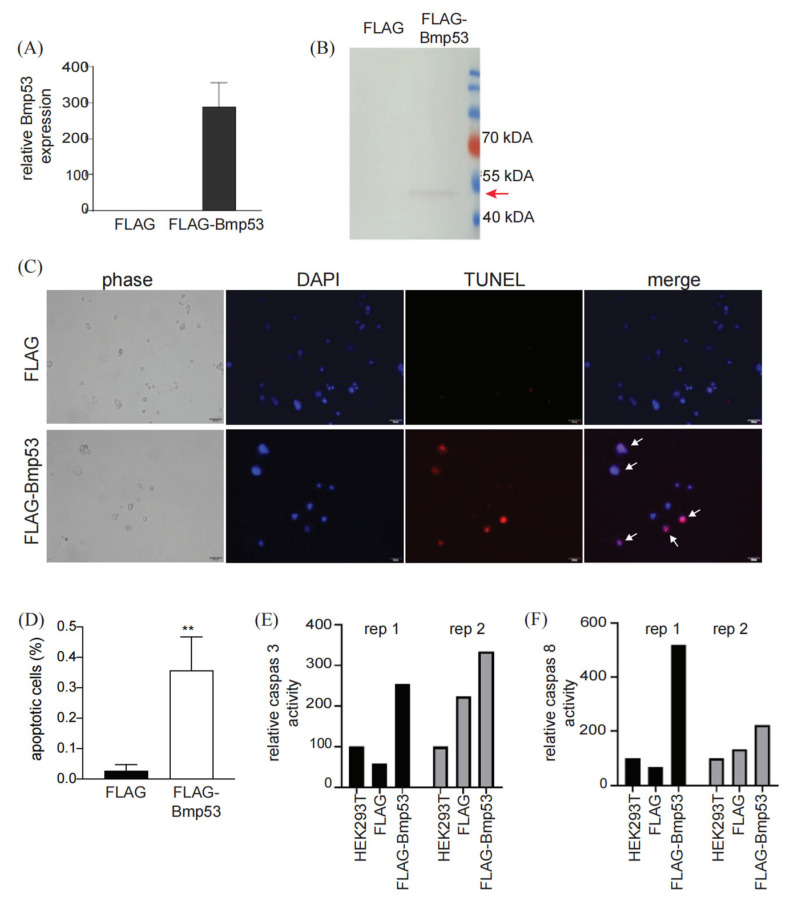
Overexpression of Bmp53 induced apoptosis in mammalian cells. (**A**) Bmp53 transcript levels in pcDNA3.1-3xFLAG-c-Bmp53 transfected and non-transfected HEK293T cells. (**B**) An anti-FLAG immunoblot of pcDNA3.1-3xFLAG-c-Bmp53 transfected and non-transfected HEK293T cells(red arrow: the location of the FLAG-Bmp53 fusion protein). (**C**) HEK293T cells transiently expressing FLAG-Bmp53 were assayed for apoptosis using a TUNEL BrightRed apoptosis detection kit. Red fluorescent signals indicate broken DNA, a hallmark of cellular apoptosis. Nuclei were counter-stained with DAPI (blue fluorescence). Co-localization of DAPI and red fluorescence is indicated by the white arrows. Scale bar = 50 μm. (**D**) The percentage of FLAG- or FLAG-Bmp53-expressing cells that were positive for the apoptotic TUNEL signal. Statistical significance determined via student *t*-test (** p < 0.01) across three transfection replicates. Signals were determined for at least 30 cells in each transfection experiment. (**E**) Overexpression of FLAG-Bmp53 in HEK293T-induced caspase 3 activity. (**F**) Overexpression of FLAG-Bmp53 in HEK293T-induced caspase 8 activity. Caspase activity was determined across duplicated experiments.

To assess Bmp53 functionality, we initially overexpressed a FLAG-tagged version of the protein in an HEK293T mammalian cell line via standard transfection protocols. As expected, Bmp53 transcripts were only detected in the FLAG-Bmp53 cells (Figure 1A), and expression of the ~45 kDa FLAG-tagged protein was confirmed (Figure 1B). To test for apoptosis in the FLAG-Bmp53 cells, we used a TUNEL BrightRed apoptosis detection system that labels the 3′ hydroxyl group of broken DNA in apoptotic cells with tetramethylrhodamine-dUTP. Unlike cells expressing the FLAG tag alone, a clear red fluorescent signal was observed in the FLAG-Bmp53 cells (Figure 1C). The signal was present in ~36% of the FLAG-Bmp53 cells and was significantly less represented (~3%) in control cells (Figure 1D), suggesting that expression of Bmp53 promoted cellular apoptosis. Since apoptotic caspases can be categorized as either initiators or effectors [1,27,28], we next assayed FLAG-Bmp53 cells for both caspase types via caspase 3 (effector) and caspase 8 (initiator) activity. At 96 hrs post transfection, we found significant increases in the activities of both enzymes in FLAG-Bmp53 cells compared to the control cells (Figure 1E,F), suggesting that Bmp53 induces late cell apoptosis through the mitochondrial pathway.

**Figure 2:**
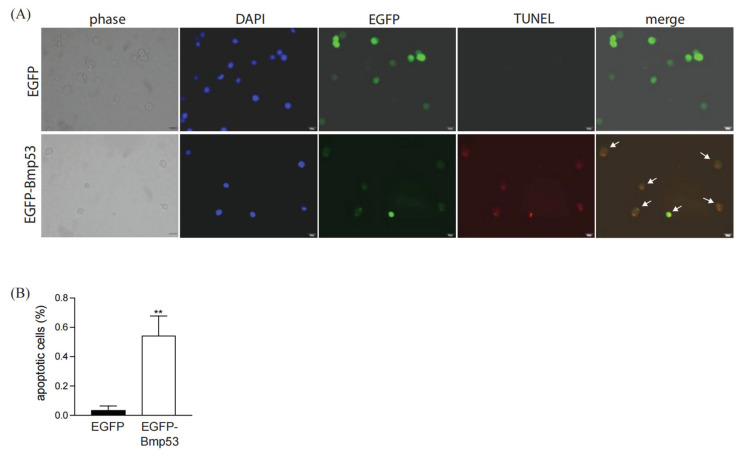
Increased cellular apoptosis in insect cells transiently expressing an EGFP-Bmp53 chimera. (**A**) BmN cells expressing the Bmp53 fusion protein were assayed for apoptosis using the TUNEL kit. Nuclei were counter-stained with DAPI (blue fluorescence). Co-localization of green (EGFP-Bmp53) and red fluorescence signals is indicated by the white arrows. Scale bar = 50 μm. (**B**) The percentage of EGFP or EGFP-Bmp53 expressing cells that were positive for the apoptotic TUNEL signal. Statistical significance determined via student *t*-test (** p < 0.01) across three transfection replicates. Signals were determined for at least 12 EGFP-positive cells in each transfection experiment.

We next examined the function of Bmp53 in BmN cells (an ovarian insect cell line) via transient expression of an EGFP-Bmp53 chimera. Similar to the mammalian cell line, red fluorescent TUNEL signals were largely restricted to cells expressing Bmp53 (Figure 2A), with significantly more EGFP-Bmp53 cells positive for the signal than control cells (Figure 2B). These results indicate that Bmp53 is capable of triggering apoptotic events through a pathway evolutionarily conserved in insect and mammalian cells.

### 3.2. Bmp53 Knockdown in B. mori Larvae Impairs Metamorphosis

**Figure 3:**
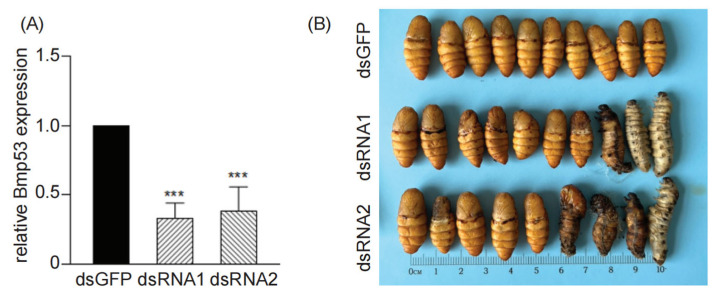
dsRNA-mediated knockdown of *Bmp53*. (**A**) Knockdown of endogenous *Bmp53* transcript levels in BmN cells following transfection with dsRNAs targeting different regions of *Bmp53* (dsRNA-1 and dsRNA-2) or GFP(*** p < 0.001). (**B**) Effects of *Bmp53* knockdown on *B. mori* pupation. Injections were performed using 6-d-old fifth instar larvae, and pupae were imaged 5 d post injection.

To assess the in vivo function of Bmp53, we designed two dsRNAs (dsRNA-1 and dsRNA-2) to target different regions of the Bmp53 coding sequence. Both dsRNAs effectively knocked down endogenous Bmp53 transcript levels in BmN cells compared to dsGFP-treated cells (Figure 3A). Given this efficacy, each of the dsRNAs was injected (0.5 µg per dsRNA) into 6-day-old fifth instar larvae, and the effects on pupation were assessed. Under normal conditions, fifth instar larvae progress to the cocoon-spinning and pupation stages on days 7–8. In the dsGFP-injected control group, all injected larvae underwent normal pupation (Figure 3B, top row). In larvae injected with the Bmp53 dsRNAs, however, a statistically significant (dsRNA1 c^2^ = 7.059, df = 1, *p* = 0.0079; dsRNA2 c^2^ = 10, df = 1, *p* = 0.0016) percentage of the larvae failed to pupate correctly. Further, we observed an elevated, but not statistically significant, number of pupae in the knockdown groups that were stunted and darker in appearance (Figure 3B), which could indicate additional pupation defects. The somewhat muted biological effect of Bmp53 knockdown may reflect biological variation and/or incomplete knockdown of the transcript (see Figure 3A), which could have left a sufficient level of protein to still drive transcription of downstream processes, albeit with less efficiency. Still, the results are consistent with Bmp53 playing a role in silkworm metamorphosis and suggest that its apoptotic function may be crucial for the tissue-generation characteristic of this development period.

### 3.3. Identification of Bmp53-Interacting Proteins

**Figure 4:**
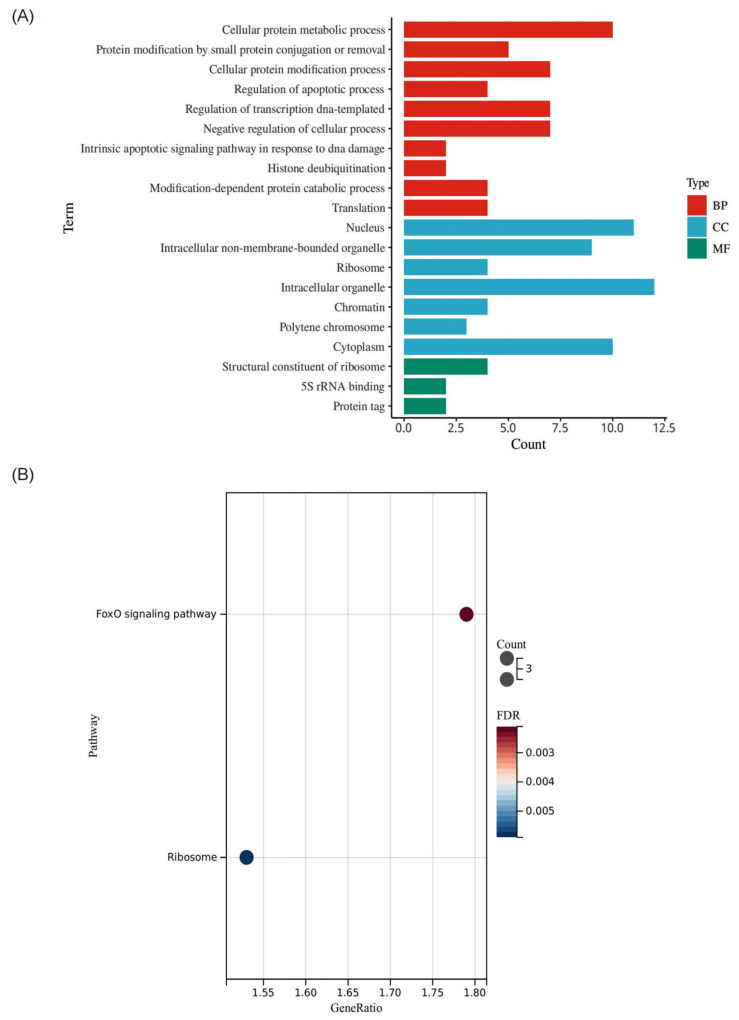
Analysis of putative Bmp53-interacting protein annotations. (**A**) Distribution of GO terms across three categories: biological process (BP), cellular component (CC), and molecular function (MF). (**B**) KEGG-based pathway enrichment. Count refers to the number of target genes annotated to a pathway. Gene Ratio = # of target genes annotated to this pathway/# of target genes annotated to all BP/MF/CC pathways.

To provide insights into the Bmp53 interactome, Y2H-Seq analysis was conducted using Bmp53 as the “bait” and cDNAs prepared from BmN cells as the “prey”. From this analysis, a total of 636 proteins were identified (Supplementary Data S1). GO enrichment analysis of the putative Bmp53-interacting proteins placed 166 proteins into one of the three GO subgroups (Figure 4A, Supplementary Data S2). Classification terms for many of the Bmp53-interacting proteins suggest roles in several growth and development processes, including regulation of the apoptotic process, histone modification, deubiquitination, and intrinsic apoptotic pathway signaling in response to DNA damage (Supplementary Data S3).

Analysis of KEGG pathways revealed that three of the Bmp53-interacting proteins were enriched for the FOXO signaling pathway and another three for the ribosome pathway (Figure 4B, Supplementary Data S2). The FOXO signaling pathway is involved in many cellular physiological events including apoptosis [29], which FOXO promotes via autophagic regulation [30,31].

In addition, ten transcription factors were among the predicted Bmp53-interacting proteins. A scan of *cis*-acting elements in the *Bmp53* gene (Supplementary Data S1 and S4) revealed recognition sites (attttgattggtccat and ataaaccaatcaaaag) for four of the transcription factors with significant network interactions (Supplementary Figure S2), such as USP1. The presence of these potentially functional *cis*-elements suggests they may directly or indirectly regulate Bmp53-mediated apoptosis.

### 3.4. Prediction of the B. mori Cell Apoptosis Pathway Regulated by Bmp53

**Figure 5:**
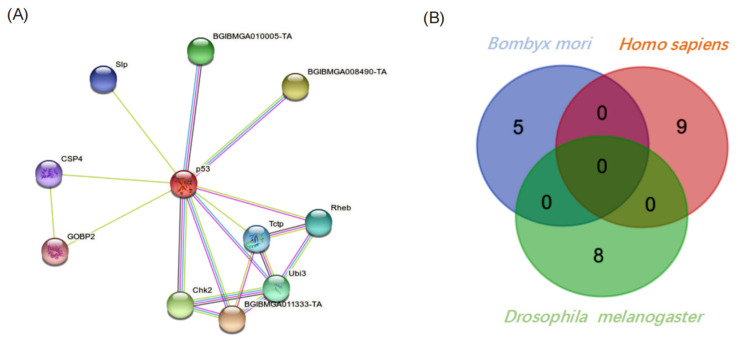
The Bmp53 apoptotic interactome. (**A**) Predicted network of protein interactions linked to Bmp53. (**B**) Venn diagram depicting the absence of overlap in p53 interacting proteins that comprise the apoptosis regulatory pathway in *Homo sapiens* and *Drosophila melanogaster*.

Based on annotations of the putative Bmp53-interacting proteins described above, we built a working model for the silkworm apoptosis network with Bmp53 as a core node. The interactome predicted by this model included five novel apoptotic regulatory proteins (Figure 5A). Although homologous with p53 in many insects, Bmp53 is phylogenetically closer to the human protein than the *D. melanogaster* protein (Supplementary Figure S3). Among the putative apoptotic regulatory interacting proteins identified in our Bmp53 Y2H-Seq screen were a number of ubiquitination regulatory proteins. This finding appears to differ significantly from the p53 regulatory networks reported for *Drosophila* and humans (Figure 5B, Supplementary Figure S4). One difference is the MDM2-p53 axis, which plays a key role in regulating cell cycle control and apoptosis. Human MDM2 contains a RING domain with ubiquitinase-binding activity that is important for protein interactions. A similar RING domain (Supplementary Figure S5) is present (residues 279–332) in the silkworm MDM2-like protein (BGIBMGA0113333-TA; BankIt Submission 2651740). Consequently, the Mdm2-like protein may be a potential ubiquitination regulator of Bmp53 in the silkworm apoptotic pathway.

## 4. Discussion

Apoptosis is a highly regulated process that leads to the death and elimination of damaged and/or malfunctioning cells, thereby playing a critical role in disease progression and tissue replacement [1,2]. Using complete genome sequences, the mechanisms underlying apoptosis in model organisms, such as humans and *D. melanogaster*, have been largely elucidated. The molecular basis of apoptosis in silkworms has yet to be developed to the same level, despite their economic importance. Silkworms undergo essential apoptosis-related remodeling of larval organs during the metamorphic changes that characterize the pupation stage [5,6,7]. To date, the functions of silkworm apoptosis-regulating proteins have been found to be similar to those in vertebrates. Here, we have shown that Bmp53 can induce apoptosis in both mammalian (HEK293T) and insect (BmN) cells (Figure 1 and Figure 2). Expanded elucidation of the silkworm pathway could provide insights into the mechanisms driving apoptosis in other lepidopterans and lead to the development of novel control strategies that impede pupal development. This latter point could have significant impacts on Chinese agroforestry, as Lepidoptera represent the main pest population. Furthermore, because insects lack the B and T cells that comprise the adaptive immune response, it is possible that the apoptotic pathway may represent an alternative defense mechanism. As such, disruption of the pathway could impact pest management strategies in general.

As an initial step for elucidating the putative Bmp53 apoptotic pathway, we sought to characterize the Bmp53 interactome (i.e., proteins interacting with Bmp53). Although Y2H approaches have traditionally been used to facilitate the identification of protein–protein interactions, the method is laborious and can be cost prohibitive [23,25]. Many technological advancements have overcome these limitations and enabled the combined application of next-generation, high-throughput sequencing technologies [24]. In this study, we applied a modified Y2H-Seq approach that omitted several expensive and time-consuming steps, greatly reducing the experimental time and cost. Using this method, we screened the Bmp53 interactome and developed a working model of the apoptotic network with Bmp53 as a core node. Several potential apoptotic regulatory interacting proteins, including ubiquitination regulatory proteins such as MDM2-like proteins, were identified, as were five new potential apoptotic regulatory proteins. Interestingly, although MDM2-p53 interactions are conserved from nematodes to vertebrates, some invertebrates, such as flies, lack MDM2 homologs [32]. MDM2, an E3 ubiquitin ligase, is typically a major negative regulator of p53 that collaborates with MDM4/MDMX to control intracellular levels of p53. In turn, MDM2 expression is modulated by a self-regulating feedback loop via p53 overexpression [33]. The MDM2-p53 axis plays a key role in regulating cell cycle control and apoptosis in mammalian species [34,35,36]. In *D. melanogaster*, several factors, such as Corp, have been identified that regulate p53 expression [33,37]. Some of these factors are homologous to MDM2 but lack E3 ubiquitin ligase activity. Unlike *D. melanogaster* and most other insects, an E3 ubiquitin ligase domain is present in the Mdm2-like Bmp53-interacting protein, which suggests that apoptotic regulation of Bmp53 may be closer to that of humans than insects. Although the importance of p53 in mammalian tumor metastasis inhibition has been gradually recognized as a function of its effect on apoptosis regulation, the underlying mechanism remains unclear [38]. Further studies on the regulation of apoptosis by Bmp53 will provide insights into the similarities and differences between the lepidopteran and mammalian regulation mechanisms and may provide new strategies for targeted intervention in pest management and/or prevention of apoptosis-associated human diseases.

## 5. Conclusions

In conclusion, our study confirmed that Bmp53 can directly induce apoptosis and that it impacts larval development during pupation. Based on our Y2H-Seq findings, we developed a model of apoptosis regulation with Bmp53 as a central node and presented data that suggest the silkworm Mdm2-like protein may be a component of the apoptotic pathway. These results provide the initial molecular foundation for elucidating apoptosis regulation in silkworms and assessing the various biological processes regulated by the Bmp53 interactome. Further study of the detailed functions of the interactome network can extend our understanding of apoptosis-related pathways in lepidoptera and identify candidate metamorphosis-associated genes for novel targeted-disruption control strategies.

## Figures and Tables

**Table 1:** Primers used.

Name	Sequence (Primer Anneal Site)	Purpose (Product Size bp)
dsGFP-Fwd	ACGTAAACGGCCACAAGTTC (start from nt 65 in ORF)	Knockdown (495)
dsGFP-Fwd-T7	GGATCCTAATACGACTCACTATAGGACGTAAACGGCCACAAGTTC	Knockdown (495)
dsGFP-Rev	TGTTCTGCTGGTAGTGGTCG (start from nt 559 in ORF)	Knockdown (495)
dsGFP-Rev-T7	GGATCCTAATACGACTCACTATAGGTGTTCTGCTGGTAGTGGTCG	Knockdown (495)
dsRNA1-Fwd	CACAAACTCTGCAGTTCCGA (start from nt 457 in ORF)	Knockdown (485)
dsRNA1-Fwd-T7	GGATCCTAATACGACTCACTATAGGCACAAACTCTGCAGTTCCGA	Knockdown (485)
dsRNA1-Rev	TTGGCTCCGATAATTTCCAG (start from nt 941 in ORF)	Knockdown (485)
dsRNA1-Rev-T7	GGATCCTAATACGACTCACTATAGGTTGGCTCCGATAATTTCCAG	Knockdown (485)
dsRNA2-Fwd	GGGCAATACAACTTCAGCGT (start from nt 232 in ORF)	Knockdown (353)
dsRNA2-Fwd-T7	GGATCCTAATACGACTCACTATAGGGGGCAATACAACTTCAGCGT	Knockdown (353)
dsRNA2-Rev	TACCAGTAGTCGGGGTCGTC (start from nt 584 in ORF)	Knockdown (353)
dsRNA2-Rev-T7	GGATCCTAATACGACTCACTATAGGTACCAGTAGTCGGGGTCGTC	Knockdown (353)
Bmp53-F	AAGGATCCATGAAACACGAAATCATGAC	Overexpression (1104)
Bmp53-R	ATACTCGAGTTCGCTGGCATGTTTCGTCG	Overexpression (1104)
Bmp53-R(CC)	ATACTCGAGccTTCGCTGGCATGTTTCGT	Overexpression (1106)
q53-F	CATCTTCACCCTGGAGAGCG	qPCR (243)
q53-R	GCTCCGATAATTTCCAGCGG	qPCR (243)

BamHI site shown in blue font; XhoI site shown in red font; cc in Bmp53-R(CC) is a spacer.

## Data Availability

Most of the analytical data are provided in the article and Supplementary Data. More original datasets used and/or analyzed during the current study are available from the corresponding author on reasonable request.

## Supplementary Materials

The following supporting information can be downloaded at: https://www.mdpi.com/article/10.3390/insects14040362/s1, Supplementary Figures S1–S5; Supplementary Data S1–S4. Figure S1: Achematic representation of vectors for Bmp53 overexpression; Figure S2: Prediction of interactions between Bmp53 transcription factors Xbp1,GFI1,TFDP1 and USF1; Figure S3: Sequence comparison (A) and phylogenetic relationship (B) of p53 protein in Bombyx mori, Drosophila melanogaster and Homo sapiens; Figure S4: Apoptosis regulation model of p53 protein in Drosophila melanogaster (A) and Homo sapiens (B); Figure S5: Analysis of MDM2-like gene in silkworm; Supplementary Data S1: Bmp53-interacting proteins identified by Y2H-Seq assay; Supplementary Data S2: GO enrichment and KEGG pathways analysis of the putative Bmp53-interacting proteins; Supplementary Data S3: Detail information of classification for the Bmp53-interacting proteins; Supplementary Data S4:Scan of cis-acting elements in the Bmp53 gene.
