# Supplementary material for: Determination of Key Components in the Bombyx mori p53 Apoptosis Regulation Network Using Y2H-Seq
Source: Insects. 2023 Apr 5;14(4):362. doi: 10.3390/insects14040362 (PMC10146131; doi:10.3390/insects14040362)
Supplement: Supplementary file 1 [file insects-14-00362-s001.zip › Supplementary Figures.pdf]

Determination of key components in the *Bombyx mori* p53 apoptosis regulation network using Y2H-Seq

Meixian Wang [1,2], Jiahao Wang [1], Ayinuer Yasen[1,2], Bingyan Fan[1,2], J. Joe Hull[3], Xingjia Shen [1,2]*

1. Jiangsu Key Laboratory of Sericultural Biology and Biotechnology, College of Biotechnology, Jiangsu University of Science and Technology, Zhenjiang 212100, Jiangsu, China
2. Key Laboratory of Silkworm and Mulberry Genetic Improvement, Ministry of Agriculture and Rural Affairs, Sericultural Research Institute, Chinese Academy of Agricultural Sciences, Zhenjiang 212100, Jiangsu, China
3. USDA-ARS Arid Land Agricultural Research Center, Maricopa, Arizona, USA

*corresponding author: Xingjia Shen shenxjsri@163.com

A)

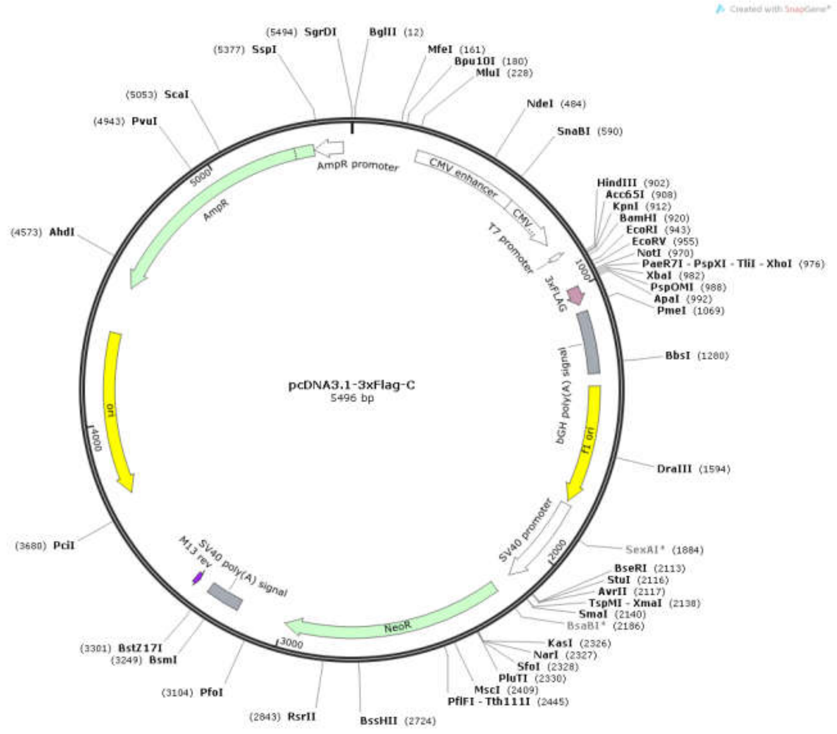

Created with SnapGene®
pcDNA3.1-3xFlag-C
5496 bp
(5494) SgrDI
BglII (12)
(5377) SspI
MfeI (161)
Bpu10I (180)
MluI (228)
(5053) ScaI
NdeI (484)
(4943) PvuI
SnaBI (590)
HindIII (902)
Acc65I (908)
KpnI (912)
BamHI (920)
EcoRI (943)
EcoRV (955)
NotI (970)
PaeR7I - PspXI - TliI - XhoI (976)
XbaI (982)
PspOMI (988)
ApaI (992)
PmeI (1069)
(4573) AhdI
BbsI (1280)
DraIII (1594)
(3680) PciI
SexAI* (1884)
BseRI (2113)
StuI (2116)
AvrII (2117)
TspMI - XmaI (2138)
SmaI (2140)
BsaBI* (2186)
KasI (2326)
NarI (2327)
SfoI (2328)
PluTI (2330)
MscI (2409)
PflFI - Tth111I (2445)
(3301) BstZ17I
(3249) BsmI
(3104) PfoI
(2843) RsrII
BssHII (2724)
AmpR promoter
AmpR
CMV enhancer
CMV
T7 promoter
3xFLAG
bGH poly(A) signal
f1 ori
SV40 promoter
NeoR
SV40 poly(A) signal
M13 rev
ori

B)

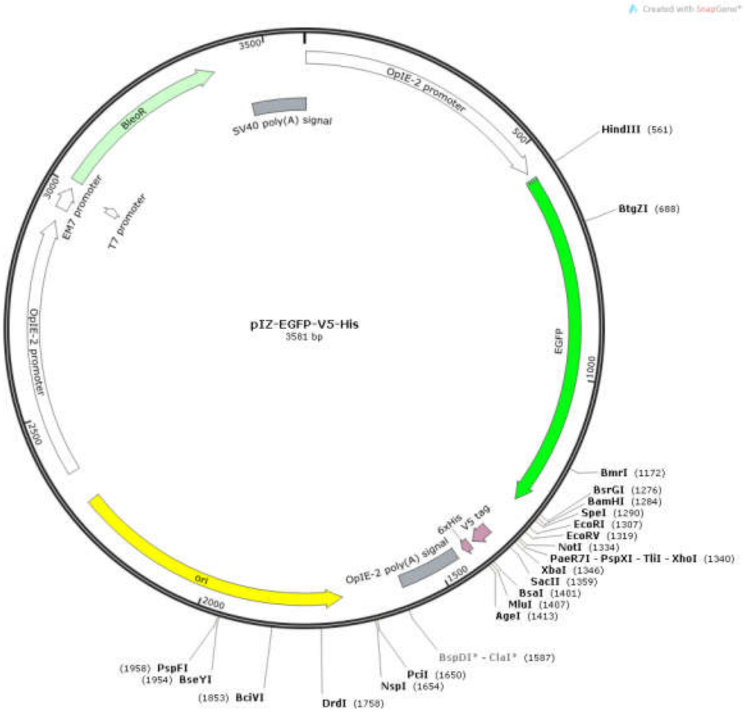

Created with SnapGene®
pIZ-EGFP-V5-His
3581 bp
HindIII (561)
BtgZI (688)
BmrI (1172)
BsrGI (1276)
BamHI (1284)
SpeI (1290)
EcoRI (1307)
EcoRV (1319)
NotI (1334)
PaeR7I - PspXI - TliI - XhoI (1340)
XbaI (1346)
SacII (1359)
BsaI (1401)
MluI (1407)
AgeI (1413)
BspDI* - ClaI* (1587)
PciI (1650)
NspI (1654)
DrdI (1758)
(1853) BciVI
(1958) PspFI
(1954) BseYI
OpIE-2 promoter
EGFP
V5 tag
6xHis
OpIE-2 poly(A) signal
ori
BleoR
EM7 promoter
T7 promoter
SV40 poly(A) signal

Supplementary Figure S1 Achematic representation of vectors for Bmp53 overexpression

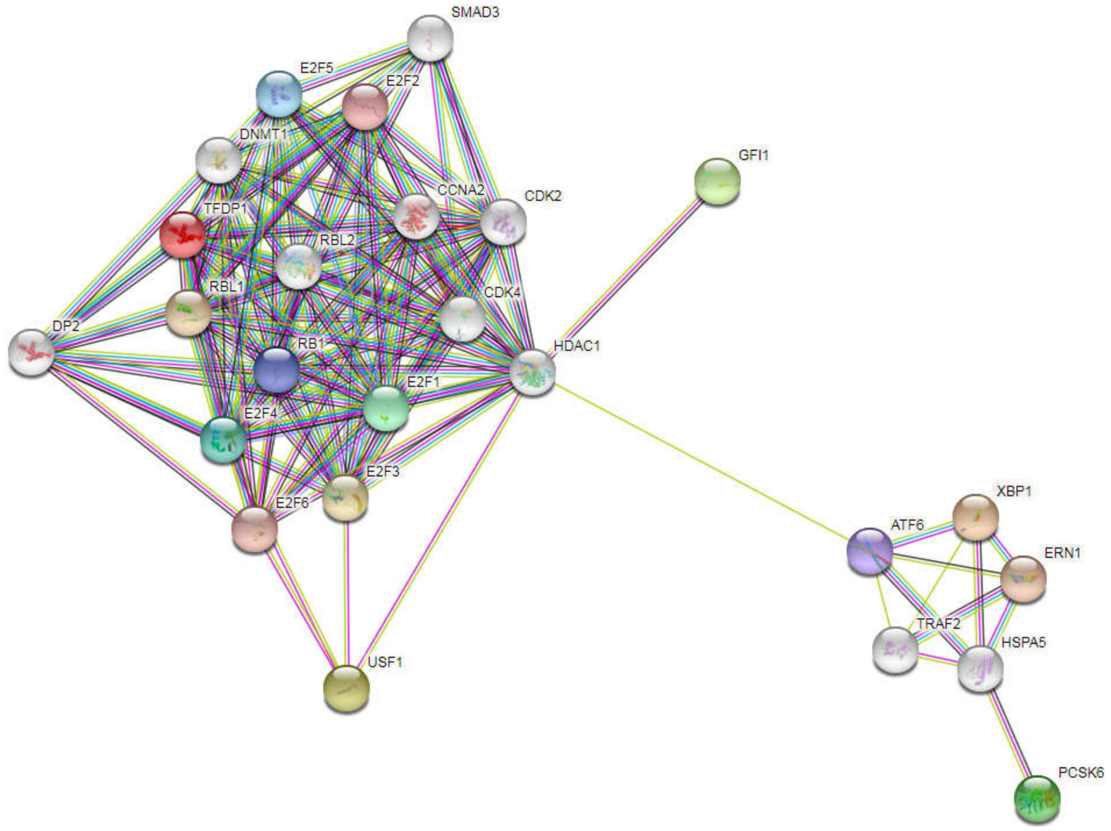

Supplementary Figure S2 Prediction of interactions between Bmp53 transcription factors Xbp1,GFI1,TFDP1 and USF1

A)

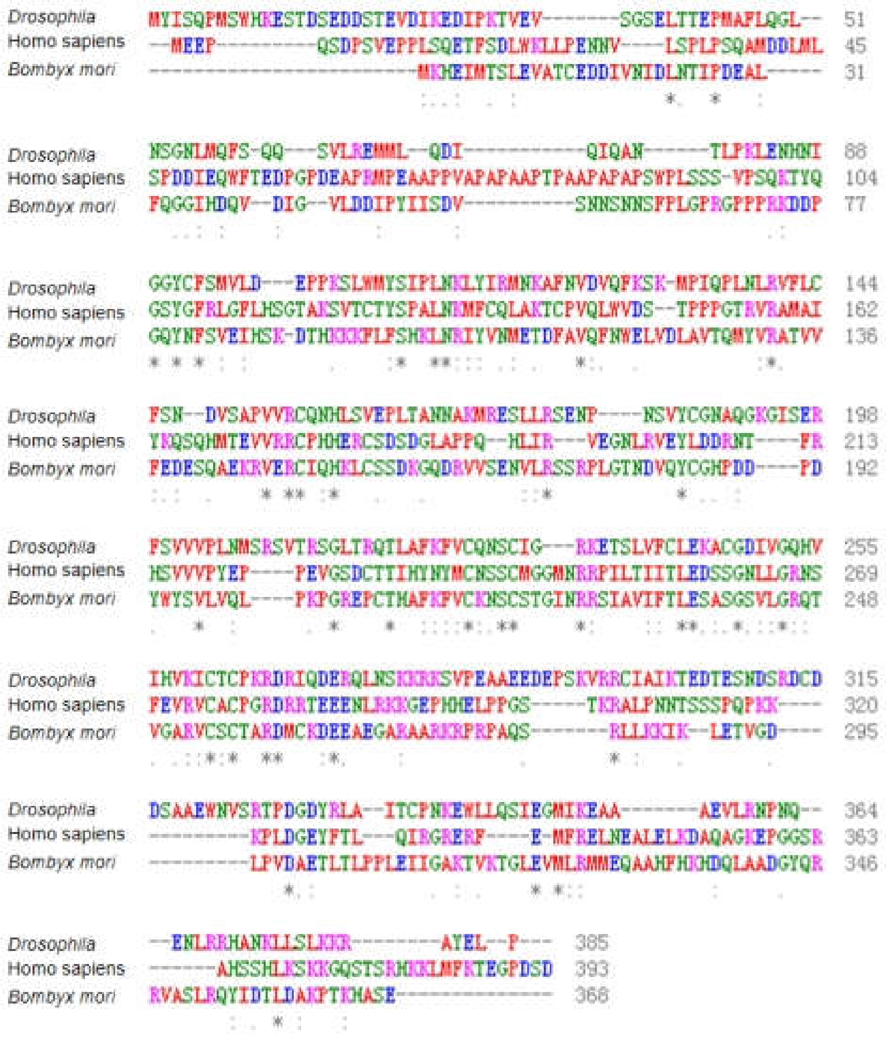

B)

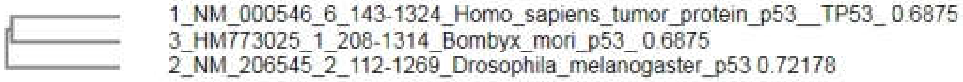

Supplementary Figure S3 Sequence comparison(A) and phylogenetic relationship(B) of p53 protein in *Bombyx mori*, *Drosophila* and *Homo sapiens*

A)

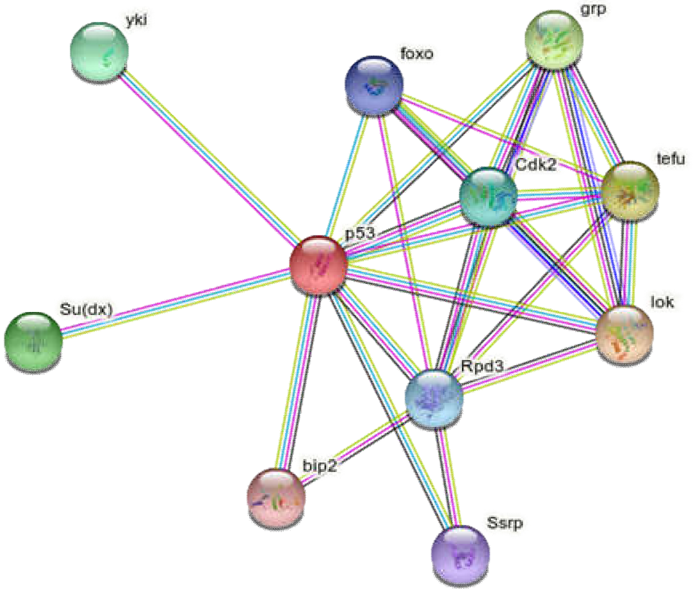

B)

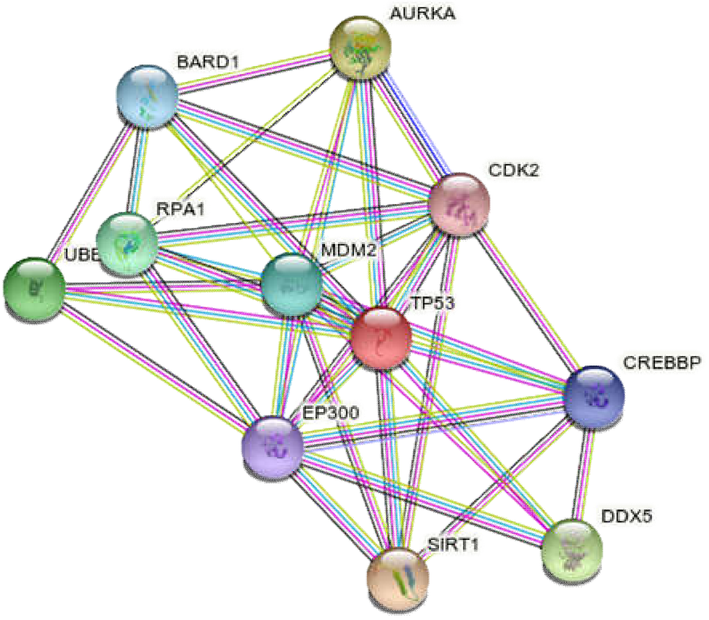

Supplementary Figure S4 Apoptosis regulation model of p53 protein in Drosophila melanogaster(A) and Homo sapiens(B)

A)

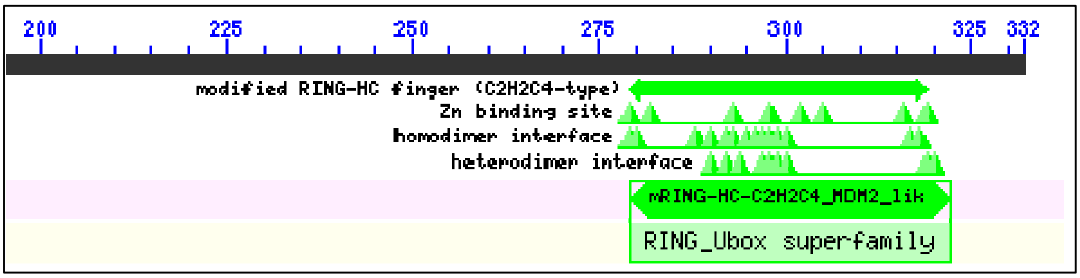

B)

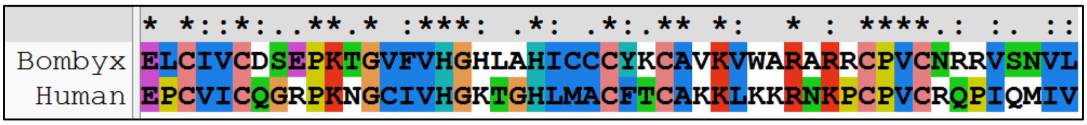

C)

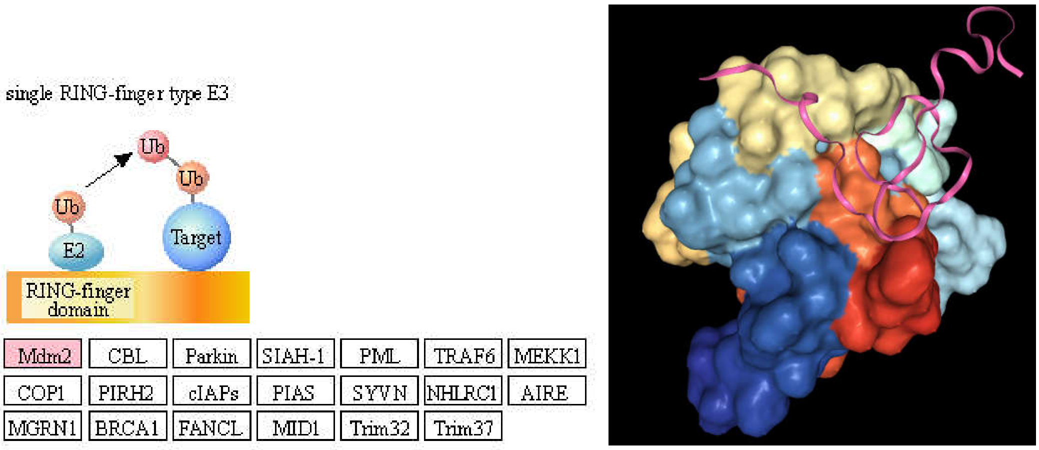

Supplementary Figure S5 Analysis of MDM2-like gene in silkworm

A) Prediction of MDM2-like protein functional domain;

B) Sequence alignment between human MDM2 and silkworm MDM2-like Ring domains;

C) Prediction of interaction between MDM2-like protein and Bmp53.
